# Self‐Standing Biohybrid Xerogels Incorporating Nanotubular Clays for Sustainable Removal of Pollutants

**DOI:** 10.1002/smll.202405215

**Published:** 2024-11-17

**Authors:** Maria Rita Caruso, Martina Maria Calvino, Pavel Šiler, Ladislav Cába, Stefana Milioto, Lorenzo Lisuzzo, Giuseppe Lazzara, Giuseppe Cavallaro

**Affiliations:** ^1^ Department of Physics and Chemistry “Emilio Segrè” University of Palermo Viale delle Science 17 Palermo 90128 Italy; ^2^ Faculty of Chemistry Institute of Materials Science Brno University of Technology Purkyňova 118 Brno 61200 Czech Republic

**Keywords:** CO_2_, environmental remediation, halloysite, hydrogels, p‐coumaric acid, xerogels

## Abstract

In this work, it is reported a scalable and systematic protocol for the preparation of xerogels based on the use of green, highly available, and low‐cost materials, i.e. halloysite nanoclay and chitosan, without the need for any expensive equipment or operational/energetic demands. Starting from colloidal dispersions, rheological studies demonstrate the formation of hydrogels with zero‐shear viscosities enhanced by ≈9 orders of magnitude and higher storage moduli. Hence, the corresponding self‐standing xerogels are prepared by a simple solvent casting method and their properties depend on the concentration of halloysite, possessing enhanced thermal stability and outstanding mechanical performances (elastic modulus and ultimate elongation of 165 MPa and 43%, respectively). The resulting biohybrid materials can be exploited for environmental remediation. High removal efficiencies are reached for the capture of organic molecules from aqueous media and the CO_2_ capture from the atmosphere is also investigated. Most importantly, the presence of an inorganic skeleton within the xerogels prevents the structure from collapsing upon drying and it allows for the control over their morphology and shape. Therefore, taking advantage of the overall features, the designed xerogels offer an attractive strategy for sustainably tackling pollution and for environmental remediation in a plethora of different domains.

## Graphical abstract

A scalable and systematic protocol for the preparation of colloidal dispersions and the corresponding hydrogels and xerogels is designed by using natural halloysite nanotubes and chitosan. Xerogels maintain the morphological properties due to the presence of nanoclays. The materials can be exploited for pollutants capture from both aqueous and gas phases, being efficient for environmental remediation.

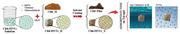

## 1 Introduction

The rapid global development has a significant impact on the environment. Industrial pollutants contaminate water, while air quality deteriorates due to anthropogenic emissions, harming ecosystems and leading to serious health conditions for the population. In the recent decades, these issues have significantly intensified, highlighting the urgent need for sustainable environmental control and innovative pollution technologies.^[^ ^1^ ^]^ Literature reports various wastewater remediation technologies, including membrane filtration, adsorption, ion exchange, and photocatalytic degradation.^[^ ^2^, ^3^, ^4^, ^5^, ^6^ ^]^ Similarly, many efforts have been made to design carbon dioxide capture technologies depending on several factors, such as cost, adsorption capacity, and stability. The main approaches can be classified as i) post‐combustion process, where the CO_2_ is captured from treated flue gas after the complete combustion in industrial plants^[^ ^7^ ^]^; ii) direct air capture (DAC), which could mitigate CO_2_ emissions from all outdoor sources^[^ ^8^ ^]^; and iii) indoor CO_2_ capture, in which the gas is captured from indoor environments, where the concentrations are significantly higher than atmospheric CO_2_ concentration.^[^ ^9^ ^]^

However, these methods come with significant drawbacks, such as expensive equipment, operational costs, and high energy requirements. Recently, the focus has shifted toward nanostructured materials due to their unique properties such as the high specific surface and higher reactivity compared to more traditional materials.^[^ ^10^, ^11^ ^]^ In general, nanoclays emerged as important resources that can act as adsorbents for pollutants.^[^ ^12^ ^]^ Halloysite, a naturally occurring nanoclay with a hollow tubular morphology,^[^ ^13^, ^14^ ^]^ shows favorable structural and chemical properties useful for many applications,^[^ ^15^, ^16^, ^17^, ^18^ ^]^ including the environmental remediation field.^[^ ^19^, ^20^, ^21^ ^]^ The main features of halloysite nanotubes (HNTs), such as dimensions and rheological behavior are influenced by the geological deposit from which they are sourced.^[^ ^22^, ^23^ ^]^ Typically, HNTs have a length of ≈1–2 µm, while the external diameter ranges from 20 to 200 nm and the internal diameter from 10 to 70 nm.^[^ ^24^ ^]^ Moreover, the surface chemistry of HNTs is noteworthy: the outer surface is composed of Si−O−Si groups, while the inner lumen surface consists of Al–OH groups.^[^ ^25^, ^26^ ^]^ This difference explains the charge separation in the pH range between 2 and 8, making halloysite ideal for carrying various negatively charged molecules encapsulated within the lumen, and positive molecules adsorbed on the outer surface.^[^ ^27^, ^28^, ^29^ ^]^ Due to its peculiar properties, halloysite proved to be an efficient adsorbent for removing organic pollutants, heavy metals, or dyes from water.^[^ ^30^, ^31^ ^]^ Unlike conventional surfactants, clays can be easily separated from the emulsions through simple centrifugation or sedimentation processes, highlighting their potential use in oil recovery.^[^ ^32^ ^]^ Biological, chemical, and physical methods have been employed as treatment solutions to reduce the impact on the environment.^[^ ^33^, ^34^ ^]^ Herein, gel materials are highly effective in environmental applications, especially due to the control over structure and morphology.^[^ ^35^, ^36^, ^37^ ^]^ Among natural materials employed for wastewater treatment, chitosan, a cationic polysaccharide, is widely recognized as a good adsorbent for various organic pollutants and metal ions.^[^ ^38^, ^39^ ^]^ However, it is still needed to enhance the adsorption capacity of chitosan, which is related to the amount of adsorbate taken up by the adsorbent per unit mass (or volume) of the adsorbent.^[^ ^40^ ^]^ A common approach to address this limitation involves the development of nanocomposites.^[^ ^41^ ^]^ For instance, composite hydrogels with chitosan and halloysite nanotubes have been successfully used to absorb methylene blue and malachite green from water solutions but also heavy metal ions.^[^ ^42^, ^43^ ^]^ However, the design of chitosan‐based dried gels displays some issues. According to literature, as a general trend, aerogels and cryogels prepared via supercritical CO_2_ (sc‐CO_2_, beyond its critical point: 73.8 bar and 31.5 °C) and freeze‐drying (liquid N_2_) methods, respectively, possess higher porosity and surface area compared to the corresponding xerogels, due to a more controlled drying step which avoids the collapse of the gel network.^[^ ^44^, ^45^, ^46^ ^]^ Nonetheless, in the specific case of chitosan‐based materials, it is reported that dried gels show poor textural properties, especially for cryogels and xerogels. In the latter case, the collapse of pore walls upon vacuum drying results in film‐like materials with smooth surfaces, which can be detrimental to their applications.^[^ ^46^, ^47^ ^]^

The main purpose of this work is to develop a simple and scalable protocol for the preparation of eco‐compatible chitosan/HNTs hydrogels and for their easy conversion into xerogel materials that maintain their structural network without any loss of morphological properties. In light of this, novel biohybrid materials can be exploited for the active capture and removal of pollutants from aquatic environments and the atmosphere.

**Figure 1:**
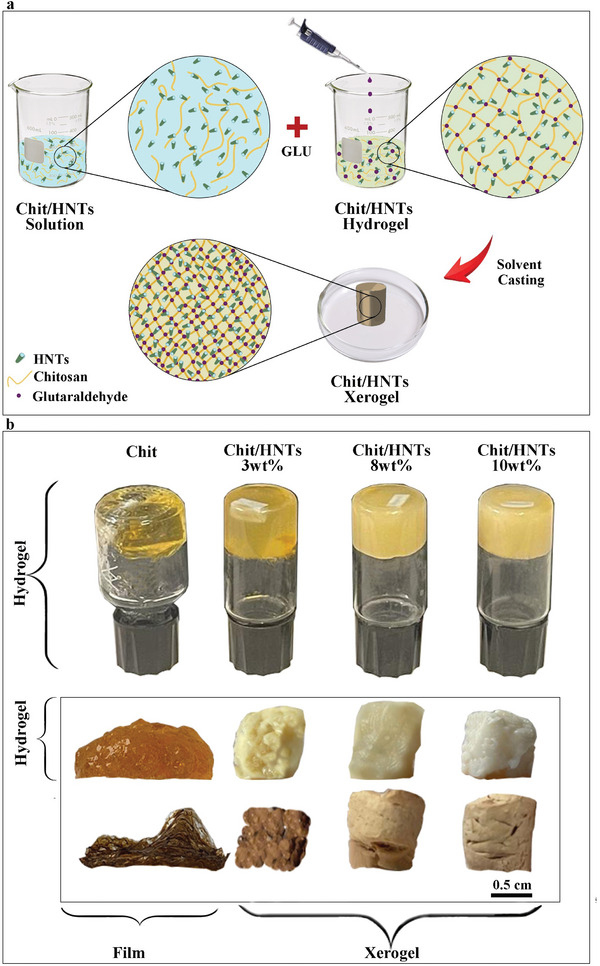
a) Schematic illustration of Chit/HNTs/GLU preparation. b) Macroscopic images of Chit and Chit/HNTs hydrogels and xerogels with increasing concentration of halloysite nanotubes.

As illustrated in **Figure** **1**a, colloidal dispersions of chitosan with increasing amounts of halloysite were prepared and glutaraldehyde was added as a cross‐linking agent for the formation of eco‐friendly hydrogels. Further solvent casting of the materials led to the preparation of xerogel systems, without any need for sc‐CO_2_ or liquid N_2_, displaying peculiar physico‐chemical properties that are crucial for environmental‐related applications. In particular, experimental characterization showed that the rheological properties depend on the addition of both the crosslinking agent and halloysite nanotubes, which are responsible for a shift from a liquid‐like behavior to a gel system. In the xerogels, the nanoclay addition results in improved thermal stability and mechanical properties. More importantly, the presence of an inorganic skeleton is pivotal, since the materials can be dried without collapsing but maintain their shape when hydrogels are converted to xerogels.

Then, the biohybrid materials were applied to capture and remove organic species from aqueous media thanks to their active surface and structural properties. The p‐coumaric acid, here employed as a representative model pollutant, belongs to the class of phenolic derivatives which are challenging to be biologically degraded as they are toxic to microorganisms and therefore inhibit the biological treatment of waste waters.^[^ ^48^ ^]^ The degradation of p‐coumaric acid present in olive oil mill wastewater allows to obtain more easily biodegradable molecules, with lower toxicity.^[^ ^49^ ^]^ It is worth noting that this phenolic compound displays two dissociation equilibrium constants (i.e. pK_a1_ = 4.70, pK_a2_ = 9.90).^[^ ^50^ ^]^ Therefore, at pH> pK_a1_ the organic molecule is bearing a negative charge due to the deprotonation of the carboxylic group to carboxylate and, as a consequence, the rising of electrostatic interactions with chitosan polymeric chains and the occurrence of ions exchange mechanism can hold a certain importance. Similar considerations can be extended to metal ions.^[^ ^38^ ^]^ Furthermore, the designed materials displayed practical utilization in air treatment, showing good efficiency in the adsorption of carbon dioxide under a flow of CO_2_ 60% v/v. Besides the simple preparation, which does not require either expensive apparatus or high operational/energetic costs, the control over structure is worth consideration. The possibility to tailor the final structure of the materials paves the ground for their use by enlarging the application domains from water bodies and reservoirs to pipelines, plant systems, and/or equipments with various dimensions and shapes, thus endowing them as promising candidates for real‐world applications in the future in both environmental applications and for the capture and conversion of waste products into added value molecules.

## 2 Results and Discussion

### 2.1 Conversion from Colloids to Gels: Macroscopic Observations

As shown in Figure 1a, the preparation of chitosan/HNTs gel systems (hydrogels and xerogels) from aqueous colloidal systems was carried out by using glutaraldehyde as a crosslinker. The composition of the gels was varied by changing the concentration of halloysite up to 10 wt.%, while the chitosan amount was constant at 1 wt.%. Specific details on the preparation protocol are presented in the Experimental Section. The samples will be identified as Chit/HNTs_C for colloidal dispersions, Chit/HNTs_H for hydrogels, and Chit/HNTs_X for xerogels, each of them followed by the amount of halloysite, i.e. 3–8–10 wt.%. The same applies to chitosan without HNTs additions, which will be referred to as Chit_C and Chit_H for colloids and hydrogels. It is worth noting that the xerogel based only on chitosan could not be prepared. In this case, the designed protocol led to the development of a film material rather than a proper xerogel (Figure 1b). This sample will be identified as Chit_film.

The formation of hydrogels was demonstrated through the tube inversion test, as depicted in Figure 1b. Solvent casting method under vacuum conditions was conducted until complete evaporation and it led to the formation of xerogels. Prior to further analysis and characterization, it is worth observing the overall behavior of each sample in the different steps. For instance, despite the diverse physico‐chemical properties that will be reported in the next paragraphs, all the hydrogels possess a typical gel structure that seems to be stronger and more stable after halloysite addition. However, once the xerogels are formed, the most significant differences arise. As it can be observed in Figure 1b, the xerogel made of chitosan without any clay addition displayed a film‐like macroscopic structure, which is due to the loss of morphological properties. Contrarily to it, the Chit/HNTs samples preserved their shape and 3D network as a consequence of halloysite addition. Such findings have outstanding importance because they make these materials versatile platforms to be exploited in different applications and operational ways. It is also worth noting that the color varies from orange to white and from brown to sand for hydro‐ and xero‐gels, respectively, becoming lighter as the concentration of halloysite increases from 0 to 10 wt.%.

### 2.2 Rheological Investigations of Colloidal Dispersions and Hydrogels

Rheological experiments were carried out to investigate the effect of halloysite nanotubes and the formation of hydrogels after the addition of glutaraldehyde. In particular, the viscosity properties and the rheological moduli of chitosan and chitosan/HNTs samples were analyzed by shear flow and frequency sweep measurements on both colloidal and hydrogel systems.

**Figure 2:**
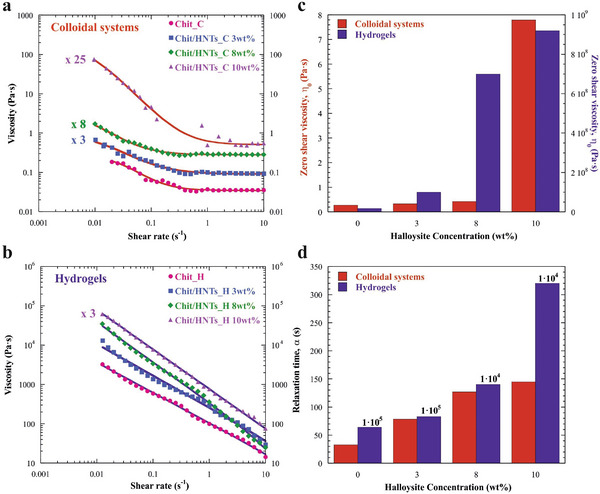
Flow experiments of colloidal dispersions and hydrogels. a) Flow curves (shear viscosity as a function of shear rate) for Chit_C and Chit/HNTs_C colloidal dispersions. b) Flow curves for Chit_H and Chit/HNTs_H hydrogels. c) Zero shear rate viscosity and d) relaxation time of colloidal systems and hydrogels. The concentration of HNTs increased from 0 to 10 wt.%. The lines in (a) and (b) represent the fitting according to the Cross model (Equation (1)).

As displayed in **Figure** **2**a,b, the flow experiments allowed to evaluate the dependence of the viscosity (η) on the shear rate (γ).

As a general trend, the viscosity decreased with the shear rate, thus indicating that all the samples behave like non‐Newtonian fluids showing shear‐thinning features.

Aimed at having more detailed insights, the η vs γ curves were fitted by using Equation (1), which describes the Cross fit model:

(1) $$\eta =\eta_{\infty }+\left(\eta_{0}-\eta_{\infty }\right)/\left(1+{\left(\alpha \cdot \gamma \right)}^{m}\right)$$

where η_0_ and η_∞_ are the zero‐shear and the infinite‐shear viscosities, α is the relaxation time, and m is the shear thinning index, which is a dimensionless parameter related to the degree of dependence of η on γ in the shear–thinning region. In particular, m can be 0 for Newtonian fluids, 1 for plastic fluids, and it can range between 0 and 1 for non‐Newtonian pseudoplastic materials, respectively.^[^ ^51^ ^]^ As shown in Figure 2a,b, the Cross model was successful in the analysis of the flow curves allowing to determine the fitting parameters for both colloidal and hydrogel systems (Figure 2c,d; Table S1, Supporting Information).

As concerns the colloidal dispersions before the addition of the crosslinking agent, the reduction of η with γ can be related to the destruction of the biopolymeric network at high shear rate. In these samples, the η_0_ values increase with the concentration of halloysite nanotubes from 0.28 Pa∙s for the chitosan acidic solution to 7.79 Pa∙s for the Chit/HNTs_C 10 wt.% colloidal dispersion (Figure 2c), highlighting a higher viscosity due to the entanglement and aggregation between nanotubes and the biopolymer adsorption on their surface. It is worth noting that the calculated values for the exponential parameter m of all samples reflect their plastic fluids behavior.^[^ ^52^ ^]^ The addition of HNTs affects the relaxation time, being α ≈2.3, 3.8, and 4.4 times higher with respect to chitosan solution for the three Chit/HNTs_C samples (Figure 2d), as a result of the reduced mobility of the polymeric chains. The presence of HNTs inhibits to a certain extent the inter‐ and intra‐ chain interactions (e.g. hydrophobic interactions, H bonds) and the diffusion of the polymer during the shear.^[^ ^53^ ^]^

As for the hydrogel samples, instead, the addition of glutaraldehyde plays a major role. Figure 2c shows that η_0_ values are enhanced by ≈9 orders of magnitude compared to those of the corresponding colloidal dispersions, in agreement with the hydrogel formation. Hence, the presence of the nanoclay did not prevent the chitosan crosslinking and the zero‐shear viscosities are higher as the concentration of halloysite increased, as observed for the liquid dispersions. In this case, the m values range from 0.77 for Chit_H up to 1 for Chit/HNTs_H 10 wt.%, evidencing that the increasing concentration of halloysite enhanced the plastic behavior. It is noteworthy represented in Figure 2d that all the hydrogels display relaxation times which are larger by several orders of magnitude compared to the colloidal systems. Since the reciprocal of α represents the critical shear rate (γ^*^), which is related to the transition from Newtonian to non‐Newtonian regime, the values were calculated to be ≈30, 13, 8, and 7×10^−3^ s^−1^ for the Chit_C and Chit/HNTs_C 3, 8 and 10 wt.% samples and 1.6, 1.2, 7.1, and 3.1×10^−7^ s^−1^ for the Chit_H and Chit/HNTs_H 3, 8 and 10 wt.% samples, respectively. The expected reduction of γ^*^ for the latter samples by ≈4 orders of magnitude is direct proof that the thinning behavior was facilitated in the hydrogels due to a greater reduction of the mobility and diffusion of the biopolymeric chains, in agreement with literature.^[^ ^51^ ^]^

**Figure 3:**
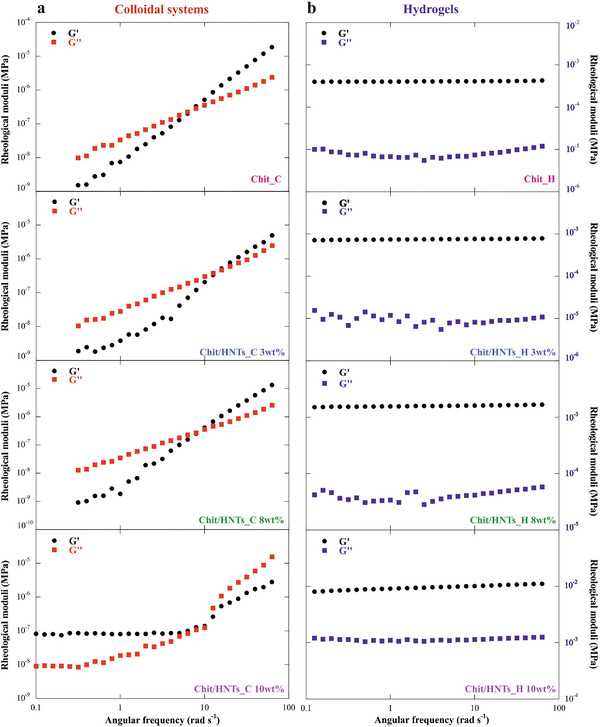
Frequency sweep experiments of colloidal dispersions and hydrogels. Storage (G’) and loss (G’’) moduli as functions of the angular frequency for a) Chit_C and Chit/HNTs_C colloidal dispersions, b) Chit_H and Chit/HNTs_H hydrogels.

The frequency sweep measurements allowed us to determine the storage (G′) and the loss (G″) moduli under variable angular frequency (ω) of the samples before and after the addition of the crosslinking agent (**Figure** **3**).

As it can be observed, the Chit_C, Chit/HNTs_C 3 wt.% and Chit/HNTs_C 8 wt.% colloidal dispersions display a crossing point between the G′ and G″ curves. The angular frequency (ω^*^) at the crossing point is related to the variations in the rheological behavior of the systems. For HNTs < 10 wt.%, the viscous component is predominant (G″ > G′) for ω < ω^*^, which means they exhibit fluid‐like behavior due to fast short‐range rearrangements. Conversely, the elastic component became predominant (G′ > G″) for ω > ω^*^, indicating a gel‐like behavior due to slow short‐range rearrangements.^[^ ^54^ ^]^ Similar results are reported in literature for colloidal suspensions based on biopolymers/clay.^[^ ^55^ ^]^ The Chit/HNTs_C 10 wt.% dispersion, instead, displays an inversion of the rheological moduli and the elastic component is predominant before the crossing point, after which it resembles a fluid system again.

Accordingly, it is possible to state that the presence of the nanoclay at high concentration improved the aggregation between the polymer and the nanotubes, providing a gel‐like behavior even before the addition of glutaraldehyde, with the crossing point indicating that the sol‐gel transition is a reversible process.^[^ ^56^ ^]^

Moreover, no crossovers can be observed in the corresponding hydrogels (Figure 3b). G′ is higher than G″ within the whole angular frequency range as expected for hydrogel systems where the elastic component overcomes the liquid behavior due to the crosslinking.^[^ ^54^ ^]^ Besides being the rheological moduli of the gel materials several orders of magnitude greater than those of the corresponding colloidal systems, they also increase with the concentration of HNTs in the hydrogels. The absence of crossing points also indicates that the gelation is not a reversible process.^[^ ^57^ ^]^ These findings represent further proofs for the formation of strong and stable 3‐D networks.

### 2.3 Properties of Chitosan/HNTs Xerogels

**Figure 4:**
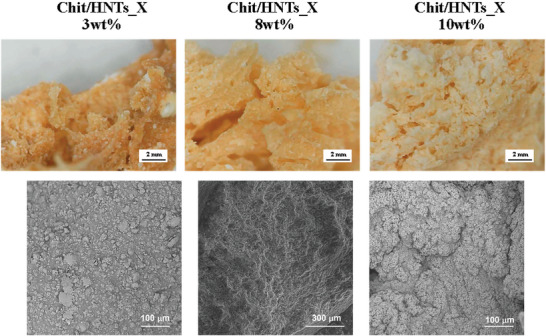
Optical and SEM images (polished cross sections) of Chit/HNTs_X xerogels with 3, 8, and 10 wt.% halloysite concentration.

One of the most remarkable achievements of this work is the design of a synthesis protocol for the easy and cost‐effective preparation of xerogels that maintain their structural properties after vacuum drying compared to the corresponding hydrogels. Optical microscopy and scanning electron microscopy (SEM) were carried out to have more insights into the morphological features of xerogels at the microscale (**Figure** **4**). As previously discussed, the xerogels have a lighter color with increasing concentrations of halloysite nanotubes. Interestingly, Chit/HNTs_X 3 wt.% sample showed clustering of clay, which resulted in white particles on the surface observed in the optical image. Conversely, the distribution becomes more homogeneous in Chit/HNTs_X 8 and 10 wt.% samples. SEM analysis confirmed these results since some clusters and aggregated nanotubes can be observed in the Chit/HNTs_X 3 wt.% sample whereas they are more homogeneously dispersed as the concentration of HNTs increased. Most importantly, both optical and SEM images showed a less dense surface for Chit/HNTs_X 10 wt.% xerogel, which displayed the presence of pores.

Aimed at assessing the textural properties of the materials, nitrogen adsorption/desorption experiments were carried out for the determination of the surface area, pore volume, pore width, and pore diameter. These results are reported in **Table** **1**.

**Table 1:** Textural properties of the Chit/HNTs_X xerogels.

Sample	S_BET_ [m^2^g^−1^]	Pore Volume [cm^3^g^−1^]	Pore Width [nm]	Pore Diameter [nm]
Chit/HNTs 3 wt.%	0.56	0.002	7.03	5.71
Chit/HNTs 8 wt.%	22.02	0.051	3.18	5.59
Chit/HNTs 10 wt.%	32.30	0.072	3.30	5.57

It can be observed that the increasing concentration of halloysite plays a major role in the overall textural properties. For instance, the surface area increases from 0.56 for the Chit/HNTs 3 wt.% up to 32.30 m^2^ g^−1^ for the Chit/HNTs 10 wt.% sample. Furthermore, although no change can be detected in either the pore width or pore diameter, the total pore volume is also profoundly influenced by the presence of the nanoclay in different amounts. Indeed, the value for Chit/HNTs 3 wt.% is equal to 0.002 cm^3^ g^−1^ and it soared by more than an order of magnitude when higher concentrations of inorganic solid are used, being 0.051 cm^3^ g^−1^ for Chit/HNTs 8 wt.% and 0.072 cm^3^ g^−1^ for Chit/HNTs 10 wt.%, which is a 36‐fold increase. It was aforementioned that, as a general trend, sc‐CO_2_‐dried aerogels and freeze‐dried cryogels display higher porosity and surface area compared to the corresponding xerogels, due to the controlled drying which avoids the collapse of the structure.^[^ ^58^ ^]^ However, as far as the chitosan‐based gels are concerned, literature reports the preparation of sc‐CO_2_ and freeze‐dried beads based on chitosan cross‐linked with sodium tripolyphosphate which possessed a surface area of ≈70–100 m^2^ g^−1^ for aerogels and which was not even detectable for cryogels.^[^ ^59^ ^]^ Similarly, Takeshita et al. prepared cryogels based on chitosan cross‐linked with formaldehyde whose surface area was not detectable.^[^ ^47^ ^]^ Li et al. also reported about the preparation of slow freeze‐dried chitosan gels cross‐linked with NaOH displaying a BET surface area of 0.5–2.9 m^2^ g^−1^ and pore volume of 0.003–0.021 cm^3^ g^−1^ and similar values are shown for gel networks based on modified chitosan, e.g. carboxymethyl chitosan, which possessed surface area equal to 0.49 m^2^ g^−1^.^[^ ^60^, ^61^ ^]^ Moreover, Chang et al. prepared different aerogels based on chitosan by changing the crosslinker and relative concentration of components and they found, for chitosan cross‐linked with glutaraldehyde (i.e. the same used in this work) a surface area of 66 m^2^ g^−1^, pore volume of 0.06 cm^3^ g^−1^ and pore diameter of ≈3 nm.^[^ ^62^ ^]^ Most interestingly, Chartier et al. prepared xerogels based on chitosan by vacuum drying and they observed a total shrinkage of the material, the absence of pores, and an undetectable surface area.^[^ ^46^ ^]^ Other works in literature report the preparation of xerogels based on chitosan with a completely smooth surface and no porosity.^[^ ^47^ ^]^ In light of this, the preparation protocol reported in this work allows for the simple design of xerogels based on chitosan that is very competitive and shows better textural properties compared to similar aerogels, cryogels, and xerogels reported in literature.^[^ ^63^ ^]^ The presence of halloysite nanotubes has major effects on the final properties of the materials, by providing them with an inorganic skeleton that avoids the structural collapse upon vacuum drying, thus resulting in self‐standing materials with certain porosity and specific surface area useful for technological applications.

**Figure 5:**
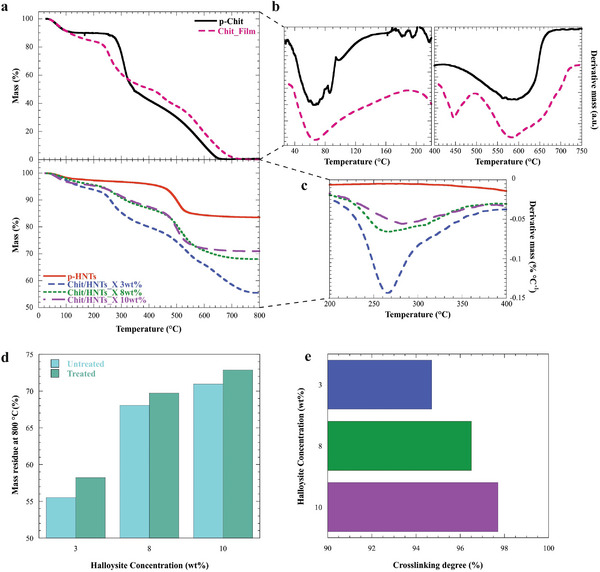
Thermogravimetric analysis of xerogels. a) Thermogravimetric and b,c) Differential Thermogravimetric curves of pure chitosan, pristine HNTs (p‐HNTs), Chit_film and Chit/HNTs_X 3, 8 and 10 wt.% xerogels. d) Mass residue of Chit/HNTs_X 3, 8, and 10 wt.% xerogels before and after treatment in a 2 wt.% acetic acid solution for 24 h. e) Cross‐linking degree was calculated using Equation (2).

The effects of halloysite addition and xerogels formation on the thermal properties of the resulting materials were investigated by thermogravimetry. The thermogravimetric (TG) curves are reported in **Figure** **5**a.

The pure chitosan displayed three different mass losses. In particular, the first one (≈150 °C) is due to the loss of water moisture. The second mass loss at 250–350 °C is related to the depolymerization and decomposition of the polymeric chains due to the deacetylation and cleavage of glycosidic bonds,^[^ ^64^ ^]^ whereas the final degradation in the 350–650 °C range reflects the pyranose ring and residual carbon decomposition.^[^ ^65^ ^]^ The Chit_film also exhibited three thermal losses and the final mass residue at 800 °C is zero for both samples due to the complete degradation of the organic matrix. The residual mass values of the hybrid materials, instead, depend on their organic/inorganic relative composition. For instance, these values are 55.5% for the Chit/HNTs_X 3 wt.%, 68.0% for the Chit/HNTs_X 8 wt.%, and 71.0% for the Chit/HNTs_X 10 wt.%. Besides, the thermogram of pristine halloysite showed the two typical degradations related to the loss of adsorbed water and the dehydroxylation of Al–OH groups, the latter occurring between 450 and 500 °C.^[^ ^66^ ^]^ Aimed at assessing the effect of the gel formation and halloysite addition on the thermal stability, differential thermogravimetric curves were analyzed. Herein, after the crosslinking of chitosan in the Chit_film, the loss of moisture is shifted to higher temperatures compared to the neat biopolymer, namely up to ≈200 °C, while the degradation of the pyranose ring occurs in the 450–700 °C range (Figure 5b). Moreover, the addition of halloysite plays a major role in the thermal stability of the composite xerogels. In fact, the DTG peak related to the depolymerization and decomposition of the chitosan chains is broader and shifted to higher temperatures as a function of the clay concentrations (Figure 5c), as a proof of improved thermal stabilization due to the interactions between the biopolymer and halloysite surface. As reported in literature, the homogenous distribution of inorganic fillers within the polymer can cause an enhancement of the thermal stability as a consequence of the barrier effect toward the volatile products and the encapsulation process within the cavity of halloysite.^[^ ^66^, ^67^ ^]^

To evaluate the crosslinking degree, TGA was also conducted after each xerogel was soaked into a 2 wt.% acetic acid solution for 24 h to solubilize the uncrosslinked polymeric fraction. As shown in Figure 5d, all the untreated samples possess lower residues at 800 °C compared to the corresponding xerogels treated with acid. This reduction is due to the loss of uncrossilinked chitosan during the reaction with CH_3_COOH.

The quantitative analysis of the residual masses allowed us to calculate the chitosan concentrations (C_Chit_) for both treated and untreated samples using the rule of mixtures.^[^ ^68^ ^]^ Then, we determined the crosslinking degree (CD) of chitosan in the composite xerogels using Equation (2):

(2) $$CD\;\left(\%\right)=100-\left(C_{Chit}^{Untreated}-C_{Chit}^{Treated}\right)$$

As shown in Figure 5e, the increase of the HNTs amount in the composite xerogel favors the chitosan crosslinking. Specifically, we determined CD results of 94.7%, 96.5%, and 97.7% for HNTs concentrations of 3, 8, and 10 wt.%, respectively. Based on this result we can state that HNTs/chitosan interactions do not prevent the cross‐linking of the biopolymeric chains by glutaraldehyde.

To characterize the interactions between chitosan and HNTs and to confirm the cross‐linking, FTIR spectroscopy was conducted (Figure S1, Supporting Information). The spectrum of pure chitosan showed a broad band in the 3000–3650 cm^−1^ range, due to the overlapping of N–H and O–H stretching vibrations, and a band in the 2800–3000 cm^−1^ range assigned to the stretching of C─H bonds.^[^ ^69^ ^]^ For what concerns the amine deformations, instead, they produced the band at 1595 cm^−1^, assigned to the N–H bending from amine overlapping the amide II vibration, and the band at 1655 cm^−1^, which is related to the C═O stretching of amide I.^[^ ^69^, ^70^ ^]^

After the cross‐linking, the 3000–3650 cm^−1^ band in the spectrum of Chit_film has lower intensity, suggesting that some hydrogen bonds involving N–H and O–H groups were destroyed and new H‐bonds were formed. Moreover, a new band appeared in the carbonyl‐amide region (Figure S1b, Supporting Information) as a split peak at 1648 cm^−1^ most likely related to the C═N bonds of imines, indicating that the material exhibited a Schiff base imine functionality as results of crosslinking, in agreement with literature.^[^ ^70^ ^]^ Figure S1c (Supporting Information) reports the spectra of pristine halloysite and the Chit/HNTs_X 10 wt.% xerogel, for comparison. Herein, the typical bands of the nanoclay can be observed. For instance, the two signals at 3695 and 3620 cm^−1^ are related to the O–H stretching vibrations of the Al_2_OH groups inside the lumen of the nanotubes.^[^ ^71^ ^]^ It is worth noting that the broad band at ≈3450 cm^−1^, related to Si–OH groups on the external surface, disappears in the xerogel thus confirming that electrostatic interactions between chitosan and HNTs take place.

**Figure 6:**
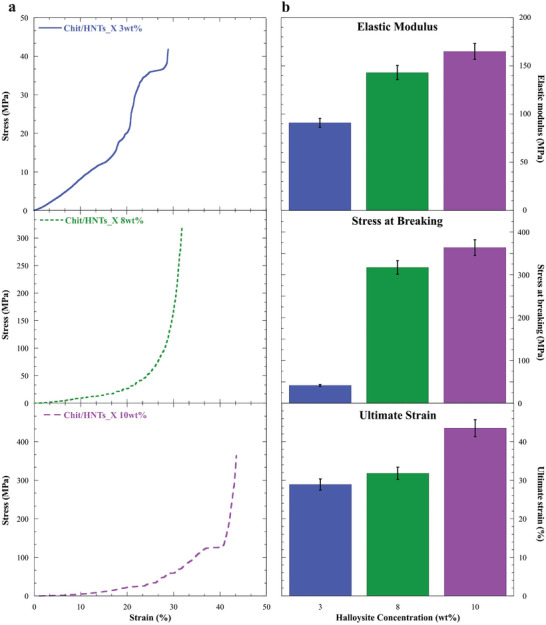
Dynamic Mechanical Analysis of xerogels. a) Stress versus strain curves and b) mechanical parameters of Chit/HNTs_X 3, 8, and 10 wt.% xerogels.

The mechanical properties of the xerogels were tested through Dynamic Mechanical Analysis (DMA) experiments in the compression mode (**Figure** **6**). The analysis of the stress versus strain curves (Figure 6a) allowed for the determination of elastic modulus, stress at breaking, and ultimate elongation, which are reported in Figure 6b. It is worth noting that the sample prepared without any halloysite addition, namely the Chit_film sample, was not tested due to its fragility upon compression.

It can be observed that all the samples have elastic properties until a certain threshold is reached. In particular, the elastic modulus showed an increasing trend with the addition of halloysite, and its values varied from 91 MPa for Chit/HNTs_X 3 wt.% to 165 MPa for Chit/HNTs_X 10 wt.%. Similarly, the maximum stress also depends on clay content, with a variation from 42 to 364 MPa for the two aforementioned samples. Concerning the ultimate elongation, the values remained nearly constant for the Chit/HNTs_X 3 and 8 wt.% samples (≈28–32%) but it soared up to 43% for Chit/HNTs_X 10 wt.% xerogel. These results showed that the mechanical performances of the xerogels are strongly dependent on the nanofiller content, whose presence provided the materials with improved elasticity and stiffness, as reported in literature for other hybrid organic/inorganic materials.^[^ ^72^ ^]^

### 2.4 Chitosan/Halloysite Xerogels for Environmental Remediation

**Figure 7:**
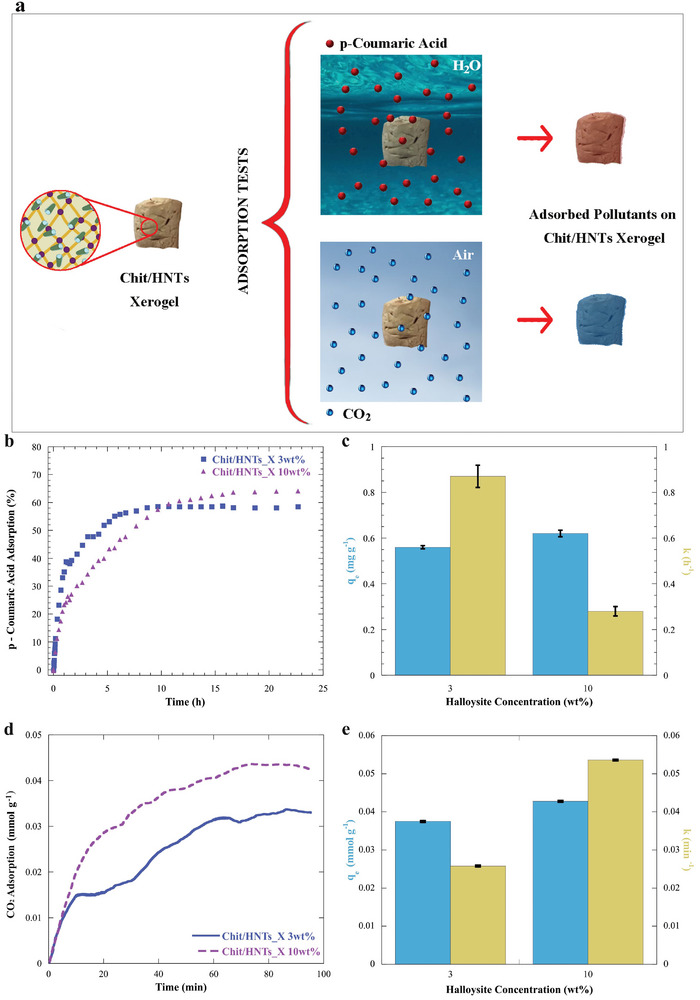
Environmental applications of Chit/HNTs xerogels. a) Schematic representation of the pollutant's adsorption on xerogels in aqueous media and air. b,c) p‐coumaric acid adsorption profiles and fitting parameters. d,e) Carbon dioxide adsorption profiles and fitting parameters (CO_2_ capture tests were performed under a flow of CO_2_ 60% v/v). The fitting parameters q_e_ and k were calculated by using Equation (3).

In the previous paragraphs, the design of the hybrid materials was conducted by a bottom‐up approach starting from the study of the colloidal systems to the evaluation of the hydrogels and, finally, focusing on their properties as xerogels. Thereafter, in order to assess their potential use for environmental purposes, the removal capacities of chitosan/HNTs xerogels toward organic molecules (coumaric acid) dispersed in aqueous media as well as the adsorption of CO_2_ gas were investigated as sketched in **Figure** **7**a.

It is worth noting that the removal of organic molecules concerns both wastewater treatment and biomass recovery and valorization, which enlarges the plethora of domains where the designed material can be used.^[^ ^73^ ^]^ The p‐coumaric acid is employed as a representative model pollutant, as it is a phenolic derivative usually found in olive oil mill wastewater and challenging to be removed and degraded. For this purpose, both the Chit/HNTs_X 3 wt.% and Chit/HNTs_X 10 wt.% xerogels were used and the analysis was carried out by recording UV–vis spectra as a function of time. The p‐coumaric acid adsorption profiles are reported in Figure 7b.

Herein, both the xerogels can remove the antioxidant species from water with an increasing trend which is followed by a plateau. In particular, the Chit/HNTs_X 3 wt.% xerogel reached the maximum uptake of ≈57% after 8.7 h and the Chit/HNTs_X 10 wt.% xerogel could retain a higher coumaric acid amount (64%) after 16.7 h. Aimed at assessing the adsorption kinetics onto the xerogels, the curves were fitted by using Equation (3):^[^ ^74^ ^]^

(3) $$q_{t}=q_{e}\;\left(1-e^{-tk}\right)\;$$

where q_t_ is the amount of absorbate per gram of absorbent at a certain time range, q_e_ represents the amount of absorbate per gram of absorbent after equilibrium conditions are reached, t is time and k is the kinetic constant. Equation (3) is a pseudo‐first‐order equation, also called Lagergren's first‐order rate equation, which is used to describe adsorption in heterogeneous systems as an adaptation from reaction kinetics in homogenous systems, which means that this model is a surface reaction model, and it suggests occupation of one active surface site by the adsorbate.^[^ ^75^ ^]^ It is noteworthy that the adsorption process is considered as an adsorbate concentration and diffusion‐controlled process.^[^ ^76^ ^]^ As far as the fitting parameters are concerned (reported in Figure 7c), q_e_ resulted to be 0.56 ± 0.01 and 0.62 ± 0.01 mg g^−1^ for the Chit/HNTs_X 3 wt.% and Chit/HNTs_X 10 wt.% xerogels, whereas the kinetics constants were 0.87 ± 0.05 h^−1^ for the former and 0.28 ± 0.02 h^−1^ for the latter, respectively. These findings suggest that Chit/HNTs_X 3 wt.% is faster in the adsorption mechanism but it can seize a lower amount of coumaric acid. Conversely, Chit/HNTs_X 10 wt.% is slower and it can capture more antioxidants. On this basis, one can state that the final amount of adsorbate and the kinetics of the capture mechanism depend on the xerogel's proper composition.

Figure 7d reports the CO_2_ adsorption of Chit/HNTs_X 3 wt.% and 10 wt.% as a function of time. Both the curves show an exponential behavior and they were fitted exploiting Equation (3), which allowed us to determine the amount of adsorbate at the equilibrium and the kinetic constant.^[^ ^77^ ^]^ The curves in Figure 7d suggest that the CO_2_ uptake follows a two‐stage process. As a matter of fact, it is possible to distinguish a linear increase that is followed by a region in which the amount of adsorbate increases slower until the equilibrium is reached. In particular, Chit/HNTs_X 10 wt.% exhibited a superior adsorption performance toward CO_2_ compared to Chit/HNTs_X 3 wt.%. It was reported elsewhere that the presence of amine groups on the chitosan structure can facilitate the acidic CO_2_ molecule to get adsorbed on the surface.^[^ ^78^ ^]^ Nevertheless, a higher contribution is given by the presence of halloysite nanotubes as confirmed by the higher values of q_e_ and k for Chit/HNTs_X 10 wt.%. Halloysite was indeed reported to be a promising candidate with good CO_2_ adsorption efficiency thanks to its structural properties.^[^ ^79^, ^80^ ^]^ Moreover, the kinetics constants are 0.026 and 0.054 min^−1^ for Chit/HNTs_X 3 wt.% and Chit/HNTs_X 10 wt.%, respectively, indicating that the adsorption process for Chit/HNTs_X 10 wt.% is faster. Therefore, these results appear to be very promising in consideration of the overall cost and ease of preparation, which should also be taken into account. For instance, literature reports different materials based on carbon nanotubes which, although showing higher adsorption performances, have a cost that is several orders of magnitude greater than those proposed in this work.^[^ ^81^ ^]^ Such findings prove that the designed xerogels represent a class of promising adsorbent materials that can be exploited for both the removal of organic pollutants from liquid/vapor phases and for the valorization and conversion of biomass to higher‐value compounds.

## 3 Conclusion

This work reports the design of biohybrid materials based on naturally occurring components, namely halloysite nanotubes (HNTs) and chitosan, that can be used for environmental applications. In particular, the study focused on a scalable and systematic protocol for the preparation of chitosan/HNTs xerogels, which were obtained by solvent casting of the corresponding hydrogels and maintained their structural network without any loss of morphological properties. The formation of xerogels can be attributed to the presence of HNTs, which confer an inorganic skeleton within the cross‐linked chitosan matrix preserving the structure upon solvent casting under vacuum conditions. Accordingly, microscopies showed that the clay nanotubes are homogeneously dispersed within chitosan. Nitrogen adsorption experiments enlightened that the vacuum‐dried gels are very competitive and show better textural properties compared to similar aerogels, cryogels, and xerogels reported in literature. Rheological experiments highlighted that halloysite favors the aggregation between biopolymeric chains in both colloidal dispersions and hydrogels. Moreover, it was observed that the HNTs filling does not prevent the gel‐like behavior of chitosan‐based hydrogel. It is worth noting that the addition of halloysite also improves the thermal stability of the composite xerogels. TGA allowed us to estimate the effect of HNTs on the chitosan cross‐linking degree, which is 94.7, 96.5, and 97.7% for halloysite concentrations of 3, 8, and 10 wt.%, respectively. These data agree with FTIR spectra, which evidenced that the chitosan/HNTs interactions do not prevent the chitosan gelation. Mechanical experiments in compression mode highlighted that larger HNTs amounts improve the elasticity and stiffness of chitosan‐based xerogels. Finally, aimed at assessing their potential use for environmental purposes, the capture of chitosan/HNTs xerogels toward p‐coumaric acid dispersed in aqueous media and the adsorption of CO_2_ from the atmosphere were investigated. Herein, the Chit/HNTs_X 10 wt.% reached the maximum coumaric acid uptake of ≈64% after 16.7 h and also exhibited a superior adsorption performance toward carbon dioxide. Compared to Chit/HNTs_X 3 wt.%, the CO_2_ adsorption efficiency increased by 10% for the xerogel with the largest HNTs amount. Such findings prove that the designed materials hold considerable promise for addressing environmental challenges associated with pollutant capture and removal from both liquid and vapor phases in a sustainable and cost‐effective manner. These aspects are crucial in the framework of sustainable development and for the valorization and conversion of biomass to higher‐value compounds.

## 4 Experimental Section

### Preparation of Chitosan/HNTs Colloidal Systems, Hydrogels and Xerogels

First, a 1 wt.% chitosan (CHIT – Sigma–Aldrich, Mw = 50–190 kg mol^−1^, deacetylation degree ≥75%) aqueous solution was prepared in 0.5 wt.% acetic acid (Sigma–Aldrich, ACS reagent, glacial, ≥99.7%) and it was magnetically stirred at 25 °C for 24 h until the complete dissolution of the polymer was reached. Then, a certain amount of halloysite nanotubes (HNTs – Sigma–Aldrich, Al_2_Si_2_O_5_(OH)_4_ · 2 H_2_O) was added as powder to 10 mL of the polymer solution, which was sonicated for 10 min and stirred for 1 h. Different colloidal systems were prepared by changing the nanoclay concentration, which namely was 0, 3, 8, and 10 wt.%, respectively. Finally, glutaraldehyde (Sigma–Aldrich, ≈50% in H_2_O, 5.6 m) at 0.5 wt.% was added as a cross‐linking agent. The dispersion was magnetically stirred until the tube inversion test confirmed the hydrogel formation. The preparation of the xerogels was carried out by solvent casting at room temperature for 12 h under vacuum conditions (P = 0.01 atm) from each hydrogel sample. The samples were identified as Chit/HNTs_C for colloidal dispersions, Chit/HNTs_H for hydrogels and Chit/HNTs_X for xerogels, each of them followed by the amount of halloysite that can be 3–8–10 wt.%, respectively. The same applies to chitosan without HNTs additions, which was referred to as Chit_C and Chit_H for colloids and hydrogels. It was worth noting that the sample Chit_X does not exist, due to its properties, and it was identified as Chit_film. Pure chitosan powder (p‐Chit) and pristine halloysite nanotubes (p‐HNTs) were also investigated for comparison.

### Materials Characterization

The rheological investigations were performed using a rheometer (Discovery HR‐1, TA Instruments) equipped with a parallel plate (40 mm diameter and 1 mm gap size). Shear‐viscosity tests were carried out in a flow ramp mode by increasing the shear rate from 0.01 to 10 s^−1^ within 60 s. The obtained flow curves (viscosity vs shear rate) were analyzed by using the Cross equation.^[^ ^51^ ^]^ In addition, frequency sweep tests were conducted with a constant strain amplitude (1%) and a variable angular frequency (from 0.01 to 10 Hz) to assess the viscoelastic properties by the determination of the storage (G’) and loss (G’’) moduli as functions of the angular frequency.

Morphological investigations of the xerogel materials were carried out by using a Digitus (DA‐70351) optical microscope and a SEM microscope (Desktop SEM Phenom PRO X PHENOM) with magnification field 160–350.000x and voltage in the range between 4.8 and 20.5 kV. Each sample was preliminarily coated with gold to avoid charging effects under an electron beam.

NOVA 2200e high‐speed gas sorption analyzer (Quantachrome Instruments) was used to study the textural properties. The samples were weighed into a measuring cell. The measuring cell was placed in a degassing station where the degassing process was carried out at 100 °C for 24 h. After cooling, the samples were placed in a measuring station. The measurement parameters were set as follows: thermal delay – 1 200 s, pressure tolerance – 0.05 Torr, equilibration time – 60 s. The adsorption and desorption isotherms were measured under the conditions of liquid nitrogen (77 K) from 0.05–0.95 of relative pressure P/P0. Obtained data were processed by NovaWin software using the BET method for the determination of the specific surface area, the Barret–Joyner–Hallenda (BJH) method for pore diameters, and the DFT method for the evaluation of pore volume and pore width.

Dynamic Mechanical Analysis (DMA) was conducted on xerogels by a DMA Q800 apparatus (TA Instruments) equipped with a compression clamp and working under a force ramp of 0.1 N min^−1^ from 0 to 10.0 N at 25.0 ± 0.5 °C. The mechanical performances were determined by the analysis of stress vs strain curves. The measurements were performed on cylindrical samples (10 mm in thickness, 0.1240 mm in diameter) which were prepared using a DMA sample cutter from TA Instrument.

Thermogravimetric analysis (TGA) was performed by using the TGA 550 Discovery Series (TA Instruments) apparatus under N_2_ flow, which was 60 cm^3^ min^−1^ for the sample and 40 cm^3^ min^−1^ for the balance, respectively. The xerogel samples (≈5 mg) were heated from room temperature up to 800 °C with a scanning rate of 20 °C min^−1^. The instrumental calibration was conducted on the basis of the Curie temperatures of standards (nickel, cobalt, and their alloys).^[^ ^82^, ^83^ ^]^

Fourier transform infrared (FTIR) studies were carried out through a Frontier FTIR spectrometer (PerkinElmer) at room temperature. The spectra were recorded in the range between 4000 and 450 cm^−1^ with a 2 cm^−1^ spectral resolution from KBr pellets with a low content (< 2 wt.%) of milled samples.

### Crosslinking Degree Evaluation

The analysis of the crosslinking degrees was carried out by thermogravimetric analysis (TGA 550 Discovery Series – TA Instruments). A certain amount (≈1g) of xerogels was weighed and soaked into a 2 wt.% acetic acid solution for 24 h. Then, the samples were dried under vacuum conditions, washed three times with water, dried again, and weighed. The crosslinking degrees were calculated as reported in the discussion paragraph.

### Pollutants Capture Tests

The adsorption of coumaric acid (p‐Coumaric acid – C_9_H_8_O_3_, Sigma–Aldrich, ≥98.0%, MW = 164 16 g mol^−1^) was studied by UV–vis spectrophotometry. In particular, the xerogels (≈0.02 g) were placed in a quartz cuvette together with 3 mL of 8 mg ml^−1^ coumaric acid solution. Spectra were recorded from 185 to 400 nm as a function of time by using a Specord S600 (Analytik, Jena, Germany). For what concerns the carbon dioxide capture, instead, the adsorption tests were conducted by using TGA equipment (TGA 550 Discovery Series – TA Instruments). Initially, each sample was equilibrated at 100 °C for 15 min to remove moisture, followed by an additional equilibration step at 30 °C for 10 min, both under nitrogen flow of 60 cm^3^ min^−1^ for the sample and 40 cm^3^ min^−1^ for the balance. The gas was then switched to CO_2_ 60% v/v (60% v/v CO_2_, 40% v/v N_2_) under isothermal conditions for 100 min and a flow of 60 cm^3^ min^−1^.

## Conflict of Interest

The authors declare no conflict of interest.

## Supporting information



Supporting Information

## Data Availability

The data that support the findings of this study are available from the corresponding author upon reasonable request.

## References

[1] A. Farinmade , O. F. Ojo , J. Trout , J. He , V. John , D. A. Blake , Y. M. Lvov , D. Zhang , D. Nguyen , A. Bose , ACS Appl. Mater. Interfaces 2020, 12, 1840. 31820921 10.1021/acsami.9b17254

[2] Y. Gao , Y. Zhu , T. Li , Z. Chen , Q. Jiang , Z. Zhao , X. Liang , C. Hu , Environ. Sci. Technol. 2021, 55, 8318. 34028264 10.1021/acs.est.1c01131

[3] R. Candeago , K. Kim , H. Vapnik , S. Cotty , M. Aubin , S. Berensmeier , A. Kushima , X. Su , ACS Appl. Mater. Interfaces 2020, 12, 49713. 33079513 10.1021/acsami.0c15570

[4] C. Jeon , K. L. Solis , H.‐R. An , Y. Hong , A. D. Igalavithana , Y. S. Ok , J. Hazard. Mater. 2020, 388, 122048. 31955026 10.1016/j.jhazmat.2020.122048

[5] L. Zhang , C. Y. Tang , C. Tang , H. Wang , J. Wang , R. Li , H. Feng , D. Yue , Small 2024, 20, 2305807. 10.1002/smll.202305807 37731008

[6] C. Chen , B. Wang , J. Xu , L. Fei , S. Raza , B. Li , Q. Zeng , L. Shen , H. Lin , Small 2024, 20, 2311427. 10.1002/smll.202311427 38733219

[7] A. Mukherjee , J. A. Okolie , A. Abdelrasoul , C. Niu , A. K. Dalai , J. Environ. Sci. 2019, 83, 46. 10.1016/j.jes.2019.03.014 31221387

[8] A. Kumar , D. G. Madden , M. Lusi , K.‐J. Chen , E. A. Daniels , T. Curtin , J. J. Perry IV , M. J. Zaworotko , Angew. Chem., Int. Ed. 2015, 54, 14372. 10.1002/anie.201506952 26440308

[9] L. R. López , P. Dessì , A. Cabrera‐Codony , L. Rocha‐Melogno , N. J. R. Kraakman , M. D. Balaguer , S. Puig , Clean. Eng. Technol. 2024, 20, 100746. 10.1016/j.scitotenv.2022.159088 36181799

[10] G. Gorrasi , V. Bugatti , V. Vittoria , Carbohydr. Polym. 2012, 89, 132. 24750614 10.1016/j.carbpol.2012.02.061

[11] W. Li , J. Li , T. Ma , G. Liao , F. Gao , W. Duan , K. Luo , C. Wang , Small 2023, 19, 2302737. 10.1002/smll.202302737 37345587

[12] G. Viscusi , E. Lamberti , G. Gorrasi , Colloids Surf. A 2022, 633, 127925.

[13] M. Liu , R. He , J. Yang , W. Zhao , C. Zhou , ACS Appl. Mater. Interfaces 2016, 8, 7709. 26967539 10.1021/acsami.6b01342

[14] K. Ariga , D. T. Leong , T. Mori , Adv. Funct. Mater. 2018, 28, 1702905.

[15] Y. Feng , D. Zhang , X. Chen , C. Zhou , M. Liu , Adv. Funct. Mater. 2024, 34, 2307157.

[16] M. Liu , R. Fakhrullin , A. Novikov , A. Panchal , Y. Lvov , Macromol. Biosci. 2019, 19, 1800419. 10.1002/mabi.201800419 30565394

[17] S. Sadjadi , M. Malmir , M. M. Heravi , F. G. Kahangi , Int. J. Biol. Macromol. 2018, 118, 1903. 30009905 10.1016/j.ijbiomac.2018.07.053

[18] Z. Jiang , S. Sun , J. Liu , X. Sun , Small 2024, 20, 2306169. 10.1002/smll.202306169 37670217

[19] A. Mohammadi , M. Kazemeini , S. Sadjadi , J. Environ. Chem. Eng. 2024, 12, 112941.

[20] Z. Su , H. Zhang , Y. Gao , L. Huo , Y. Wu , X. Ba , Chem. Eng. J. 2020, 393, 124695.

[21] A. Lo Bianco , M. M. Calvino , G. Cavallaro , L. Lisuzzo , P. Pasbakhsh , S. Milioto , G. Lazzara , Y. Lvov , Small, 2406812, 10.1002/smll.2024068. PMC11656676 39375983

[22] M. M. Calvino , G. Cavallaro , P. Pasbakhsh , G. Lazzara , S. Milioto , J. Mol. Liq. 2024, 394, 123721.

[23] L. Lisuzzo , M. Bertini , G. Lazzara , C. Ferlito , F. Ferrante , D. Duca , Appl. Clay Sci. 2023, 245, 107121.

[24] P. Pasbakhsh , G. J. Churchman , J. L. Keeling , Appl. Clay Sci. 2013, 74, 47.

[25] L. Lisuzzo , G. Cavallaro , S. Milioto , G. Lazzara , Appl. Clay Sci. 2024, 247, 107217.

[26] Y. Wang , X. Ba , B. Zhang , Y. Wang , Y. Wu , H. Zhang , J. Colloid Interface Sci. 2024, 657, 344. 38043236 10.1016/j.jcis.2023.11.173

[27] D. Fix , D. V. Andreeva , Y. M. Lvov , D. G. Shchukin , H. Möhwald , Adv. Funct. Mater. 2009, 19, 1720.

[28] L. Wang , P. Wang , X. Xue , D. Wang , H. Shang , Y. Zhao , B. Zhang , J. Colloid Interface Sci. 2024, 665, 88. 38518423 10.1016/j.jcis.2024.03.109

[29] X. Zhao , C. Zhou , Y. Lvov , M. Liu , Small 2019, 15, 1900357. 10.1002/smll.201900357 30957957

[30] G. Zeng , Y. He , Y. Zhan , L. Zhang , Y. Pan , C. Zhang , Z. Yu , J. Hazard. Mater. 2016, 317, 60. 27262273 10.1016/j.jhazmat.2016.05.049

[31] Y. Zhao , E. Abdullayev , A. Vasiliev , Y. Lvov , J. Colloid Interface Sci. 2013, 406, 121. 23806416 10.1016/j.jcis.2013.05.072

[32] T. Zhao , J. Chen , Y. Chen , Y. Zhang , J. Peng , J. Dispersion Sci. Technol. 2021, 42, 934.

[33] M. R. Zahi , W. Zam , M. El Hattab , Food Chem. 2022, 381, 132238. 35114626 10.1016/j.foodchem.2022.132238

[34] B. Micó‐Vicent , F. M. Martínez‐Verdú , A. Novikov , A. Stavitskaya , V. Vinokurov , E. Rozhina , R. Fakhrullin , R. Yendluri , Y. Lvov , Adv. Funct. Mater. 2018, 28, 1703553.

[35] Y. Zou , J. Zhao , J. Zhu , X. Guo , P. Chen , G. Duan , X. Liu , Y. Li , ACS Appl. Mater. Interfaces 2021, 13, 7617. 33538165 10.1021/acsami.0c22584

[36] P. Strachowski , M. Fronczak , E. Olechno , M. Kowalik , W. Kiciński , W. Kaszuwara , M. Bystrzejewski , New J. Chem. 2018, 42, 7073.

[37] Y. Wang , S. Oldenhof , F. Versluis , M. Shah , K. Zhang , V. van Steijn , X. Guo , R. Eelkema , J. H. van Esch , Small 2019, 15, 1804154. 10.1002/smll.201804154 30698916

[38] Y. Zhang , K. Yan , F. Ji , L. Zhang , Adv. Funct. Mater. 2018, 28, 1806340.

[39] J. Luo , A. Ji , G. Xia , L. Liu , J. Yan , Molecules 2024, 29, 1609. 38611888 10.3390/molecules29071609 PMC11013490

[40] M. Antilén , F. Amiama , M. Otaiza , F. Armijo , M. Escudey , C. Pizarro , N. Arancibia‐Miranda , J. Nanopart. Res. 2015, 17, 212.

[41] M.‐Y. Chang , R.‐S. Juang , J. Colloid Interface Sci. 2004, 278, 18. 15313633 10.1016/j.jcis.2004.05.029

[42] K. D. Nguyen , T. T. C. Trang , T. Kobayashi , J. Appl. Polym. Sci. 2019, 136, 47207.

[43] Q. Peng , M. Liu , J. Zheng , C. Zhou , Microporous Mesoporous Mater. 2015, 201, 190.

[44] H. Feng , Y. Hu , J. Liu , Q. Li , N. Chen , H. Feng , Sci. Adv. Mater. 2023, 15, 1575.

[45] S. Frindy , A. Primo , A. el kacem Qaiss , R. Bouhfid , M. Lahcini , H. Garcia , M. Bousmina , A. El Kadib , Carbohydr. Polym. 2016, 146, 353. 27112884 10.1016/j.carbpol.2016.03.077

[46] C. Chartier , S. Buwalda , H. Van Den Berghe , B. Nottelet , T. Budtova , Int. J. Biol. Macromol. 2022, 202, 215. 35033531 10.1016/j.ijbiomac.2022.01.042

[47] S. Takeshita , A. Sadeghpour , W. J. Malfait , A. Konishi , K. Otake , S. Yoda , Biomacromolecules 2019, 20, 2051. 30908038 10.1021/acs.biomac.9b00246

[48] R. Jarboui , H. Baati , F. Fetoui , A. Gargouri , N. Gharsallah , E. Ammar , Environ. Technol. 2012, 33, 951. 22720420 10.1080/09593330.2011.603753

[49] J. M. Monteagudo , M. Carmona , A. Durán , Chemosphere 2005, 60, 1103. 15993158 10.1016/j.chemosphere.2004.12.063

[50] A. Benvidi , A. Dadras , S. Abbasi , M. D. Tezerjani , M. Rezaeinasab , R. Tabaraki , M. Namazian , J. Chine. Chem. Soc. 2019, 66, 589.

[51] G. Cavallaro , M. R. Caruso , S. Milioto , R. Fakhrullin , G. Lazzara , Int. J. Biol. Macromol. 2022, 222, 228. 36155783 10.1016/j.ijbiomac.2022.09.170

[52] L. Lisuzzo , G. Cavallaro , G. Lazzara , S. Milioto , Carbohydr. Polym. Technol. Appl. 2023, 6, 100380.

[53] B. Huang , M. Liu , Z. Long , Y. Shen , C. Zhou , Mater. Sci. Eng., C 2017, 70, 303. 10.1016/j.msec.2016.09.001 27770895

[54] M. H. Kim , Y. W. Lee , W.‐K. Jung , J. Oh , S. Y. Nam , J. Mech. Behav. Biomed. Mater. 2019, 98, 187. 31252328 10.1016/j.jmbbm.2019.06.014

[55] J. Zlopasa , B. Norder , E. A. B. Koenders , S. J. Picken , Carbohydr. Polym. 2016, 151, 144. 27474553 10.1016/j.carbpol.2016.05.055

[56] T. Wu , N. Kummer , K. J. De France , S. Campioni , Z. Zeng , G. Siqueira , J. Dong , G. Nyström , Carbohydr. Polym. 2021, 251, 117021. 33142582 10.1016/j.carbpol.2020.117021

[57] W. Argüelles‐Monal , F. M. Goycoolea , C. Peniche , I. Higuera‐Ciapara , Polym. Gels Networks 1998, 6, 429.

[58] L. Thoni , B. Klemmed , M. Georgi , A. Benad , S. Klosz , A. Eychmüller , RSC Adv. 2020, 10, 2277. 35494579 10.1039/c9ra09631k PMC9048761

[59] R. M. Obaidat , B. M. Tashtoush , M. F. Bayan , R. T. Al Bustami , M. Alnaief , AAPS Pharm. Sci. Tech. 2015, 16, 1235. 10.1208/s12249-015-0312-2 PMC4666257 25761387

[60] C. Li , Q. Dang , Q. Yang , D. Chen , H. Zhu , J. Chen , R. Liu , X. Wang , RSC Adv. 2022, 12, 21041. 35919839 10.1039/d2ra01875f PMC9301543

[61] W. Luo , Z. Bai , Y. Zhu , RSC Adv. 2018, 8, 13370. 35542515 10.1039/c7ra13064c PMC9079812

[62] X. Chang , D. Chen , X. Jiao , J. Phys. Chem. B 2008, 112, 7721. 18543985 10.1021/jp8011359

[63] N. Hammi , N. Couzon , T. Loiseau , C. Volkringer , A. El Kadib , S. Royer , J. Dhainaut , Mater. Today Sustain. 2023, 22, 100394.

[64] M. Ziegler‐Borowska , D. Chełminiak , H. Kaczmarek , J. Therm. Anal. Calorim. 2015, 119, 499.

[65] I. Corazzari , R. Nisticò , F. Turci , M. G. Faga , F. Franzoso , S. Tabasso , G. Magnacca , Polym. Degrad. Stab. 2015, 112, 1.

[66] L. Lisuzzo , G. Cavallaro , S. Milioto , G. Lazzara , Appl. Clay Sci. 2020, 185, 105416.

[67] M. Makaremi , P. Pasbakhsh , G. Cavallaro , G. Lazzara , Y. K. Aw , S. M. Lee , S. Milioto , ACS Appl. Mater. Interfaces 2017, 9, 17476. 28481104 10.1021/acsami.7b04297

[68] M. M. Calvino , L. Lisuzzo , G. Cavallaro , G. Lazzara , S. Milioto , J. Environ. Chem. Eng. 2022, 10, 108594.

[69] G. Lawrie , I. Keen , B. Drew , A. Chandler‐Temple , L. Rintoul , P. Fredericks , L. Grøndahl , Biomacromolecules 2007, 8, 2533. 17591747 10.1021/bm070014y

[70] J. D. Schiffman , C. L. Schauer , Biomacromolecules 2007, 8, 594. 17291083 10.1021/bm060804s

[71] F. Ferrante , M. Bertini , C. Ferlito , L. Lisuzzo , G. Lazzara , D. Duca , Appl. Clay Sci. 2023, 232, 106813.

[72] L. Lisuzzo , G. Cavallaro , S. Milioto , G. Lazzara , Appl. Clay Sci. 2021, 213, 106231.

[73] S. Thakur , A. Verma , V. Kumar , X. Jin Yang , S. Krishnamurthy , F. Coulon , V. K. Thakur , Fuel 2022, 309, 122114.

[74] J. P. Vareda , J. Mol. Liq. 2023, 376, 121416.

[75] W. Rudzinski , W. Plazinski , J. Phys. Chem. B 2006, 110, 16514. 16913785 10.1021/jp061779n

[76] W. Plazinski , W. Rudzinski , J. Phys. Chem. C 2009, 113, 12495.

[77] M. Niu , H. Yang , X. Zhang , Y. Wang , A. Tang , ACS Appl. Mater. Interfaces 2016, 8, 17312. 27315143 10.1021/acsami.6b05044

[78] S. M. Rafigh , A. Heydarinasab , ACS Sustainable Chem. Eng. 2017, 5, 10379.

[79] K. Jeamjumnunja , O. Cheycharoen , N. Phongzitthiganna , S. Hannongbua , C. Prasittichai , ACS Appl. Nano Mater. 2021, 4, 3686.

[80] K. Ramadass , G. Singh , K. S. Lakhi , M. R. Benzigar , J.‐H. Yang , S. Kim , A. M. Almajid , T. Belperio , A. Vinu , Microporous Mesoporous Mater. 2019, 277, 229.

[81] F. Raganati , F. Miccio , P. Ammendola , Energy Fuels 2021, 35, 12845.

[82] I. Blanco , G. Cicala , A. Latteri , G. Saccullo , A. M. M. El‐Sabbagh , G. Ziegmann , J. Therm. Anal. Calorim. 2017, 127, 147.

[83] I. Blanco , L. Abate , F. A. Bottino , P. Bottino , Polym. Degrad. Stab. 2014, 102, 132.

