# Supplementary material for: Self‐Standing Biohybrid Xerogels Incorporating Nanotubular Clays for Sustainable Removal of Pollutants
Source: Small. 2024 Nov 17;21(3):2405215. doi: 10.1002/smll.202405215 (PMC11753492; doi:10.1002/smll.202405215)
Supplement: Supplementary file 1 — Supporting Information [file SMLL-21-2405215-s001.docx]

**Supporting Information**

**Self-Standing Biohybrid Xerogels Incorporating Nanotubular Clays for Sustainable Removal of Pollutants**

*Maria Rita Caruso, Martina Maria Calvino, Pavel Šiler, Vladislav Cába, Stefana Milioto, Lorenzo Lisuzzo,* Giuseppe Lazzara, Giuseppe Cavallaro*

M.R. Caruso, M.M. Calvino, S. Milioto, L. Lisuzzo, G. Lazzara, G. Cavallaro

Department of Physics and Chemistry “Emilio Segrè”

University of Palermo

Viale delle Science 17, 90128, Palermo, Italy
E-mail: lorenzo.lisuzzo@unipa.it (corresponding author)

P. Šiler, V. Cába

Faculty of Chemistry

Institute of Materials Science

Brno University of Technology

Purkyňova 118, Brno 61200, Czech Republic

**Table S1.** Fitting parameters obtained from the analysis of the flow curves based on Cross model for the colloidal systems and the hydrogels.

|  | **η_0_ (Pa·s)** | **α (s)** | **m** | **R^2^** |
| --- | --- | --- | --- | --- |
| **Colloidal systems** |  |  |  |  |
| Chit_C | 0.280 | 33.08 | 1.54 | 0.982 |
| Chit/HNTs_C 3 wt% | 0.328 | 78.44 | 1.15 | 0.982 |
| Chit/HNTs_C 8 wt% | 0.423 | 127.0 | 1.34 | 0.989 |
| Chit/HNTs_C 10 wt% | 7.796 | 144.7 | 1.49 | 0.963 |
| **Hydrogels** |  |  |  |  |
| Chit_H | 1.8 ∙ 10^7^ | 6.4 ∙ 10^6^ | 0.77 | 0.997 |
| Chit/HNTs_H 3 wt% | 1.0 ∙ 10^8^ | 8.3 ∙ 10^6^ | 0.81 | 0.993 |
| Chit/HNTs_H 8 wt% | 7.0 ∙ 10^8^ | 1.4 ∙ 10^6^ | 1.02 | 0.998 |
| Chit/HNTs_H 10 wt% | 9.2 ∙ 10^8^ | 3.2 ∙ 10^6^ | 1.00 | 0.998 |

**FTIR:**

*Chitosan vibrations:* C-O stretching at 1076 cm^-1^, antisymmetric C-O-C and C-N stretching at 1155 cm^-1^, CH_3_ and CH_2_ deformation and bending at 1384 and 1424 cm^-1^.

*Halloysite vibrations:* O–H bending of water at 1638 cm^-1^, perpendicular Si–O stretching at 1114 cm^-1^, in-plane Si–O stretching at 1030 cm^-1^, deformation of inner hydroxyl groups at 911 cm^-1^ and Al–O–Si deformation at 536 cm^-1^.


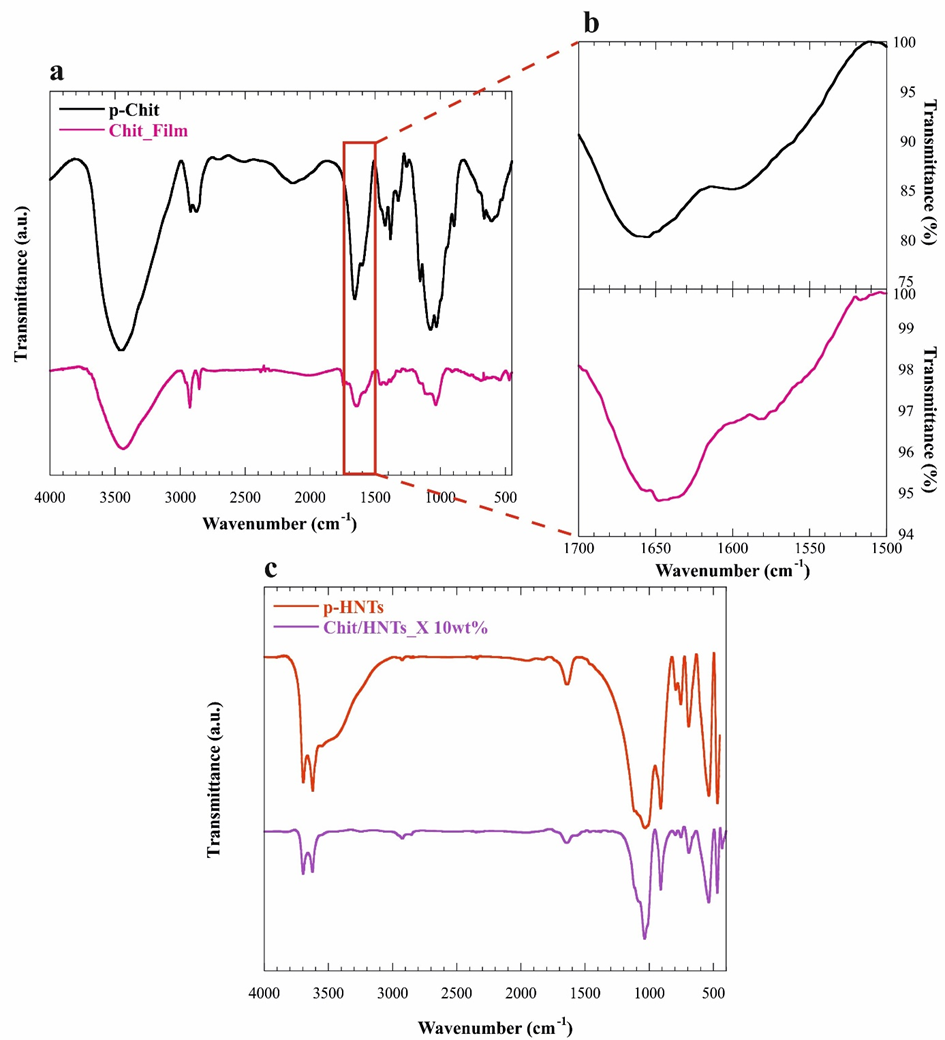


**Figure S1.** FTIR spectroscopy measurements. (a) FTIR spectra of pure chitosan (p-Chit) powder and Chit_film and (b) 1500-1700 cm^-1^ range inset. (c) FTIR spectra of pristine HNTs and Chit/HNTs_X 10wt% xerogel.

**Table S2.** Mechanical parameters of Chit/HNTs_H 3, 8 and 10wt% hydrogels resulting from the analysis of stress vs strain curves.

| Sample | Elastic Modulus [MPa] | Stress at Breaking [MPa] | Ultimate Elongation [%] |
| --- | --- | --- | --- |
| Chit/HNTs 3wt% | 91 ± 5 | 41.8 ± 2.1 | 28.9 ± 1.4 |
| Chit/HNTs 8wt% | 143 ± 7 | 317.2 ± 15.9 | 31.8 ± 1.6 |
| Chit/HNTs 10 wt% | 165 ± 9 | 363.6 ± 18.2 | 43.5 ± 2.2 |
